# Cementitious composite materials for thermal energy storage applications: a preliminary characterization and theoretical analysis

**DOI:** 10.1038/s41598-020-69502-0

**Published:** 2020-07-30

**Authors:** Luca Lavagna, Davide Burlon, Roberto Nisticò, Vincenza Brancato, Andrea Frazzica, Matteo Pavese, Eliodoro Chiavazzo

**Affiliations:** 1 Department of Applied Science and Technology, Politecnico di Torino, 10129 Torino, Italy; 2 Department of Energy, Politecnico di Torino, 10129 Torino, Italy; 3 CNR, Istituto di Tecnologie Avanzate per l’Energia “Nicola Giordano” ITAE, 98126 Messina, Italy; 4 Present Address: Independent Researcher, Via Borgomasino 39, 10149 Torino, Italy

**Keywords:** Chemical engineering, Mechanical engineering

## Abstract

The lack of robust and low-cost sorbent materials still represents a formidable technological barrier for long-term storage of (renewable) thermal energy and more generally for Adsorptive Heat Transformations—AHT. In this work, we introduce a novel approach for synthesizing cement-based composite sorbent materials. In fact, considering the number of available hygrosopic salts that can be accommodated into a cementitious matrix—whose morphological properties can be also fine-tuned—the new proposed in situ synthesis paves the way to the generation of an entire new class of possible sorbents for AHT. Here, solely focusing on magnesium sulfate in a class G cement matrix, we show preliminary morphological, mechanical and calorimetric characterization of sub-optimal material samples. Our analysis enables us to theoretically estimate one of the most important figures of merit for the considered applications, namely the energy density which was found to range within 0.088–0.2 GJ/m^3^ (for the best tested sample) under reasonable operating conditions for space heating applications and temperate climate. The above estimates are found to be lower than other composite materials in the literature. Nonetheless, although no special material optimization has been implemented, our samples already compare favourably with most of the known materials in terms of specific cost of stored energy. Finally, an interesting aspect is found in the ageing tests under water sorption-desorption cycling, where a negligible variation in the adsorption capability is demonstrated after over one-hundred cycles.

## Introduction

Despite the widespread abundance of renewable heat sources especially at low and medium temperature (e.g. solar thermal energy) its extensive exploitation remains challenging due to the intermittency issue. Thermal energy storage (TES) technologies are essential for moving towards reliable and competitive renewable energy sources in the next future. More specifically, thermochemical and sorption based energy storage has attracted much attention in the scientific community due to a strong potential to achieve large energy density combined to negligible losses even for long-term applications. Sorption TES materials for low and medium temperatures have the potential to achieve storage densities from 6 to 10 times higher than common sensible TES medium^1^. A considerable number of studies have been focusing on development of seasonal solar TES specifically for the residential sector^2,3^. However, the above issue turned out to be a formidable engineering challenge and, among other problems, the development of low-cost and robust sorbents has revealed one of the most critical roadblock^4^.

For instance, salt hydrates are largely studied for their high energy density. However, they are prone to deliquescence and cracks formation also leading to low cyclability. On the other side, solid microporous sorbents, such as zeolites or silica gels, are characterized by a high level of hydrothermal stability, with higher power outputs and cyclability, at the expenses of lower energy densities and higher cost^5–7^.

Therefore, in the recent years, in an attempt of overcoming the above issues, we have witnessed a growing interest towards composite sorption materials which are formed by at least two components. The one serving as the host matrix, while the other working as the active sorption material (the salt hydrate). In case of hydrophilic sorbents (such as zeolites or silica gels) used as host matrices, they may also give a contribution to the sorption heat released by the composite^3^. The above composite materials are also referred to as ‘salt inside porous matrix’ materials (CSPM)^8^. Clearly, the host matrix must be highly porous, so as to host a considerable amount of salt crystals. At the same time, the matrix porosity must not be filled completely with the active material, otherwise the water vapour flow reveals problematic and the solution formed inside the pores can leak out due to the volume expansion^9^.

As far as the synthesis of CSPMs is concerned, matrix impregnation with saline aqueous solutions is the most widely used method^8^ and it can be performed by two different approaches, namely dry and wet impregnation^10^. Although many studies have been reported in the literature on the performance of various CSPMs for TES applications (the interested reader can found a comprehensive overview in^3^), it is fair to say that the technological maturity level of CSPMs for sorption heat storage is still very low especially as far as stability and cost is concerned. Some recent activities, below described, demonstrate that the current research trends focus on identifying optimal CSPMs composition, configurations and synthesis routes in order to maximize the achievable energy storage density as well as cycling stability to help this class of materials quickly achieving a maturity level for real-scale applications. Liu et al.^11^ presented an innovative concept of composite sorbent for open sorption TES applications, based on a low-cost mesoporous matrix, siliceous shale, and LiCl (9.6 wt%) as embedded salt. The main innovation related to the idea of shaping the composite as a honeycomb structure, whose channels could allow the flow of the treated air, in order to perform a direct heat and mass exchange with the material. The experimental results demonstrated superior performance compared to pure CaCl_2_ considered as benchmark. Furthermore, also appreciable stability up to 250 cycles was reported. Nevertheless, possible issues regarding carryover of salt during the open sorption TES operation were neglected. Recently, Zhang et al.^12^ proposed a novel class of matrices, namely, mesoporous alumina, to obtain mechanically and hydrothermally stable composite sorbents, using LiCl as embedded salt. The results confirmed the reliable sorption behaviour of the synthesized samples, even if the achievable storage density was limited by the poor mesoporous volume characterizing the selected matrix. Furthermore, the cost was not considered as parameter for the selection of alumina as matrix, which could represent a barrier towards the scale-up of this composite. A novel approach, both in terms of synthesis process and matrix selection was firstly proposed by Brancato et al.^13^. The concept relies on the use of polymeric foams as hosting matrix in which the salt is embedded directly in situ during the foaming process. In such a way the grains of salt should be evenly distributed directly inside the macro-pores of the structure. Moreover, thanks to the permeability of the selected polymer towards the water vapour, the salt is embedded in a closed porosity, which can prevent any salt solution leakage during the operation. The first results reported in^13^, employing MgSO_4_ 7H_2_O showed promising results in terms of dehydration reactivity, compared to the bulk salt. Nevertheless, the amount of embedded salt was always lower than expected, probably due to issues during the proposed synthesis process, which should be optimized. Similar approach was proposed, using SrBr_2_ 6H_2_O as embedded salt, by Calabrese et al.^14^, proving the applicability of the process to different salts, but also highlighting wide room for improvements, due to the difficulties in obtaining sufficiently homogeneous and reliable samples. Interesting results were reported on a novel composite based on impregnation of SrBr_2_ 6H_2_O into a mesoporous Metal Organic Framework (MOF), referred as MIL-101(Cr), by D’Ans et al.^15^. The flexible MOF structure was able to embed up to 63 wt% of salt, achieving very high TES density, i.e. 233 kWh/m^3^. Particularly, the sorption capacity measured on the composite resulted closer to the one achievable by the pure salt. Two possible explanations were proposed by the authors, either the salt inside the pores is absorbing more water than the expected 6 molecules, thus forming a solution, or the MOF itself is physically adsorbing water molecules, thus enhancing the overall sorption ability of the composite. Furthermore, preliminary cycling analysis showed quite stable behaviour of the composite up to 10 cycles. Despite the interesting analysis performed, it is clear how the cost of the pristine materials proposed still represents a barrier for further development of this solution.

Starting from the analysis of the recent development activities reported in the literature and above reported, here, we introduce the concept of cement-based composite materials directly obtained by mixing the cement paste with salt hydrates, thus exploiting the natural porosity formation in cement and a salt crystal precipitation within the pores. Below, this new approach will be referred to as in situ synthesis. After an initial characterization of porous cement and the selection of a reference pure cement material, the samples obtained by in situ synthesis are characterized from the morphological and calorimetric point of view. In addition, hydro-thermal stability has been also assessed with the ageing of the cement-based composites tested up to 300 water adsorption cycles.

Finally, based on the equilibrium water adsorption measurements, we computed ideal thermodynamic cycles possibly driven by solar heat up to 150 °C and we estimated one of the most relevant figures of merit, namely the (material based) energy density.

## Results

**Figure 1:**
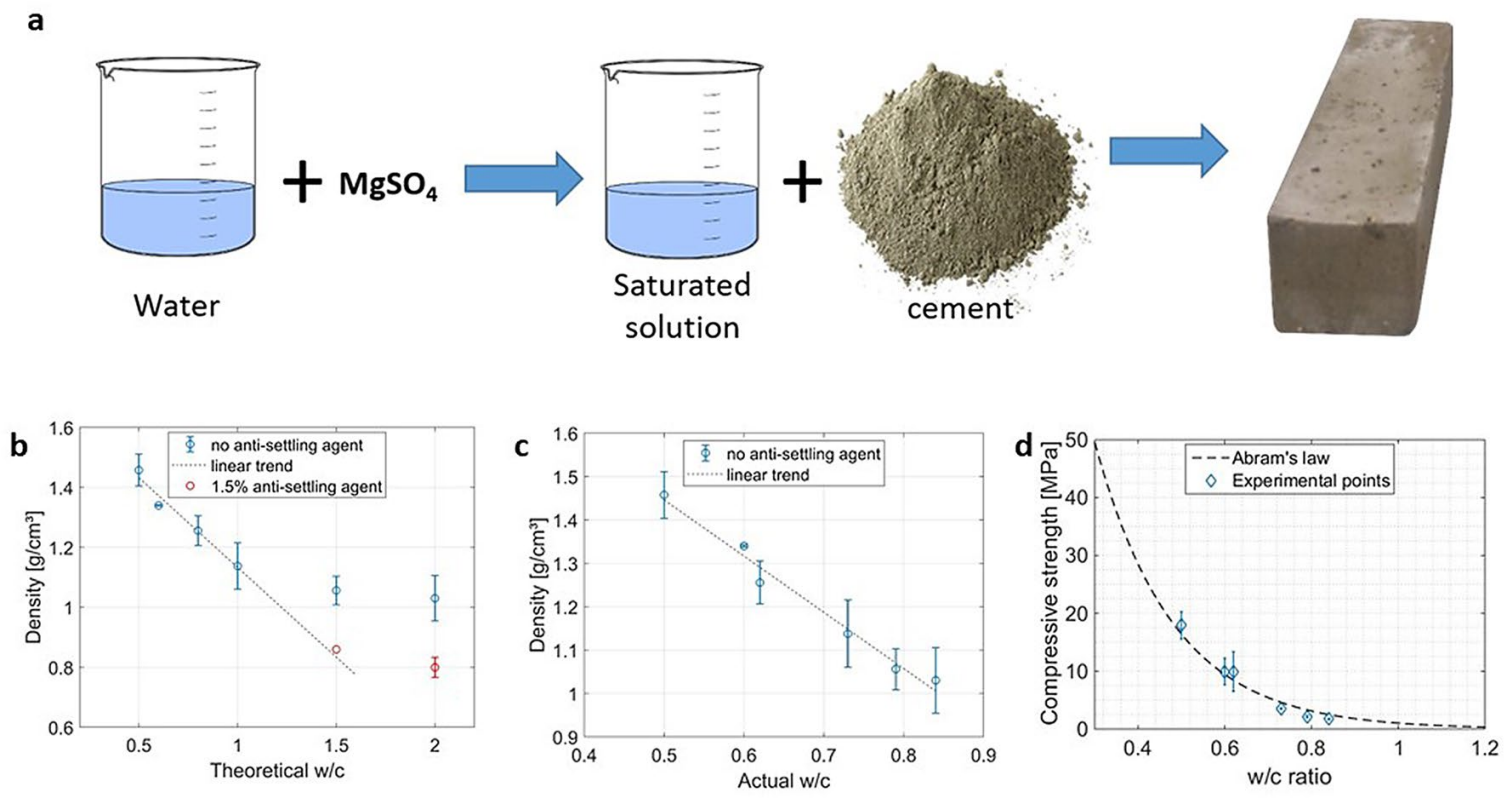
(**a**) Preparation scheme for composite samples containing magnesium sulphate (**b**) Samples density dependence on theoretical *w*/*c* ratio, (**c**) on actual *w*/*c* ratio and (**d**) the correlation between cement compressive strength and *w*/*c* ratio fitted with the Abrams’ Law.

The overall aim of this work is to synthesize and characterize a new composite material for adsorption heat storage applications. The main idea is to adopt a widespread, easily accessible and low-cost material, such as cement paste, as a possible matrix host for salt hydrates. In fact, upon hydration, cement is known to naturally develop a significant degree of porosity^16^, which can be conveniently controlled by acting on the water-cement ratio. At a given hydration degree, the porosity of the hardened cement paste is directly proportional to the water-to-cement ratio used^17^ while mechanical properties are inversely proportional^18^. Interestingly, unlike more traditional approaches, the use of cement-based host matrices enables to both fine tune porosity (by controlling the water-to-cement ratio) and to introduce an innovative in situ synthesis. The latter synthesis consists in a direct production of sorbents by properly mixing the cement paste with a salt-containing aqueous solution (instead of pure water). The sample preparation scheme is shown in Fig. 1 and details are described in the methodological section below.

### Morphological and mechanical analysis

To evaluate the total porosity of the cement matrix, the simplest method is to evaluate the density of the hardened cement paste after drying to evaporate all the unreacted water. The effect of the water-to-cement ratio on density is shown in Fig. 1b. Theoretically, the density should decrease continuously with the *w*/*c* ratio. Nevertheless, when exceeding $w/c = 1$ the density stops following a decreasing linear trend, and flattens on values near 1 g/cm^3^. This is due to a well-known phenomenon referred to as *bleeding*, leading to the separation of excess water from the cement paste. The latter phenomenon can be partly countered with the use of an anti-settling agent, as shown by the two red points in Fig. 1b. Due to the phenomenon of bleeding, thus, the initial *w*/*c* ratio is not anymore a good measure of the porosity of the samples, and must be substituted with the actual *w*/*c* ratio, which does not include the water separated from the cement paste. In Fig. 1c, we report the density values against the actual *w*/*c* ratio. In line with the theoretically predicted density of the hydrated cement (dotted line in Fig. 1c), a linear decrease is observed. Following the theory of Collepardi^19^, the stoichiometric *w*/*c* ratio needed for the complete hydration of cement is 0.23, but since water is adsorbed on the nano-sized grains of calcium silicate hydrates, the effective *w*/*c* ratio needed for the complete hydration of cement is 0.42. Knowing that the density of cement powder $d_c$ is approximately 3.1 g/cm^3^, it is possible to calculate the density of hydrated cement according to the following equation:

1 $$\begin{aligned} d_{hc} = \frac{1 + \frac{w^*}{c}}{\frac{1}{d_c}+\frac{w}{c}} \end{aligned}$$

with $d_{hc}$ being the density of hydrated cement. A density of water $d_w=1$ is considered for simplicity, *w*/*c* is the actual water-to-cement ratio, and w*/*c* is equal to 0.23 considering that all the free and absorbed water evaporates (i.e. due to drying at high temperature). Since the measured density values are well fitted from the calculated values, it is also possible to calculate the porosity with the following equation:

2 $$\begin{aligned} p_{hc} = \frac{\frac{w}{c}-\frac{1.14}{d_c}+0.19}{\frac{1}{d_c}+\frac{w}{c}} \end{aligned}$$

where $p_{\textit{hc}}$ is the porosity of hydrated cement, and 1.14 comes from the theory reported in^19^.

In the case of composite samples obtained by in situ synthesis, it is difficult to accurately measure the density value due to the fast setting of the salt-containing cement paste, so it can be calculated by the following equation:

3 $$\begin{aligned} d_{hc-salt} = \frac{1+\frac{w^*}{c}+\frac{s}{c}}{\frac{1}{d_c}+\frac{\frac{w}{c}+\frac{s}{c}}{d^*_{sol}}} \end{aligned}$$

where *s*/*c* is the weight ratio between salt and cement and $d_{sol}^*$ is the density of the solution of salt into water, that for the case under study has a value of 1.263 g/cm^3^^20^.

Similarly, the porosity value can be calculated by the following equation:

4 $$\begin{aligned} p_{hc-salt} = \frac{\frac{\frac{w}{c}+\frac{s}{c}}{d^*_{sol}}-\frac{1.14}{d_c}+0.19}{\frac{1}{d_c}+\frac{\frac{w}{c}+\frac{s}{c}}{d^*_{sol}}} \end{aligned}$$

Finally, the salt content in the composite can be calculated from the equation:

5 $$\begin{aligned} f_s=\frac{\frac{s}{c}}{1.23+\frac{s}{c}} \end{aligned}$$

where $f_s$ is the fraction of salt in the composite with respect to the total composite weight.

The calculated density of the in situ composite samples is $\rho _{comp}= 1.13$ g/cm^3^ while the porosity is 58.7%, higher than the reference cement samples (52.8%). The calculated salt content of the composite is 20.8%, with the *s*/*c* ratio being 0.323.

Samples of cement were subjected to compression tests, with the results shown in Fig. 1d. As expected, the compressive strength decreases with the actual *w*/*c* ratio. The results were fitted with a generalized version of the popular Abrams’ Law^18^, that is shown as a dotted line in Fig. 1d.

**Figure 2:**
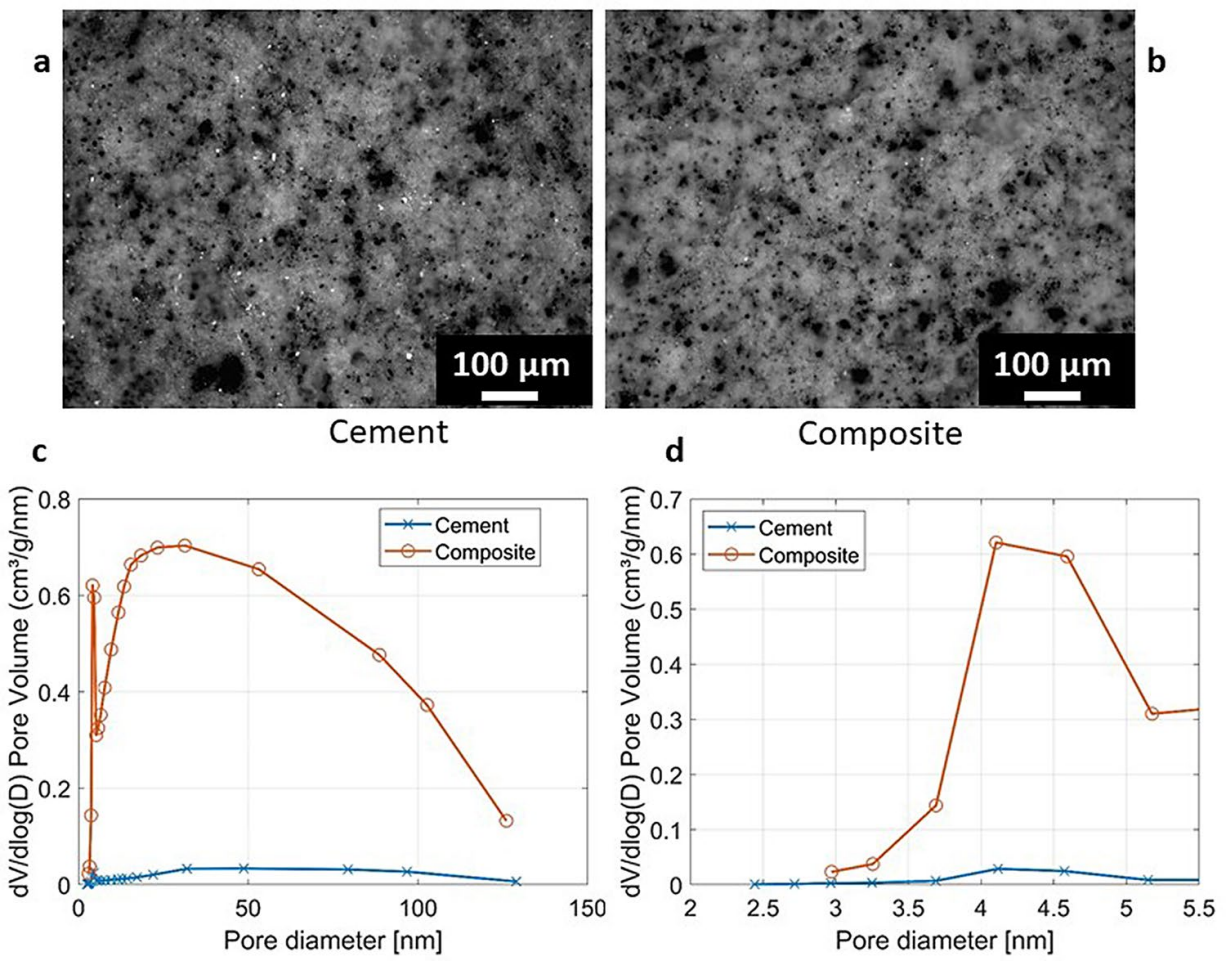
Top: Cross-section surface micrographs of cement (**a**) and composite sample (**b**). Bottom: Comparison between different pore size distribution of cement and composite sample, overall distribution (**c**) and detail for pore dimensions between 2 and 5.5 mm (**d**).

Optical microscopy images obtained on polished cement surfaces are reported In Fig. 2a,b. Those images show that both cement and composite samples present a high porosity fraction, and that the composites present larger pores than the pure cement. The same porosity increase is shown by gas-volumetric N_2_ adsorption. All the cement samples have fairly high surface areas, ranging between about 10 and 20 m^2^/g, demonstrating the presence of a fine sub-micrometric porosity, that was further evaluated by applying the Barrett–Joyner–Halenda model (BJH)^21,22^, which is the method used to determine the pore distribution and the volume of pores in mesoporous materials. Since BJH method outcomes differ whether the adsorption or the desorption branch of the hysteresis loop is considered, in this work the desorption branch was taken into account. The pore distribution, shown in Fig. 2c,d, demonstrates that the volume of small pores increases in the in situ composite samples with respect to the pure cement. The higher adsorption of the composite sample is in accordance with its higher calculated porosity and with the sorption isotherms.

### Preliminary calorimetric analysis

In a typical water sorption thermal energy storage system, sorbent hydration occurs using water vapour. However, in order to assess to which extent the in situ synthesised samples could develop temperature lifts, we conducted our first calorimetric tests by hydrating the cement-based composites with liquid water. Details on the testing procedure are reported below in the Methods section.

**Figure 3:**
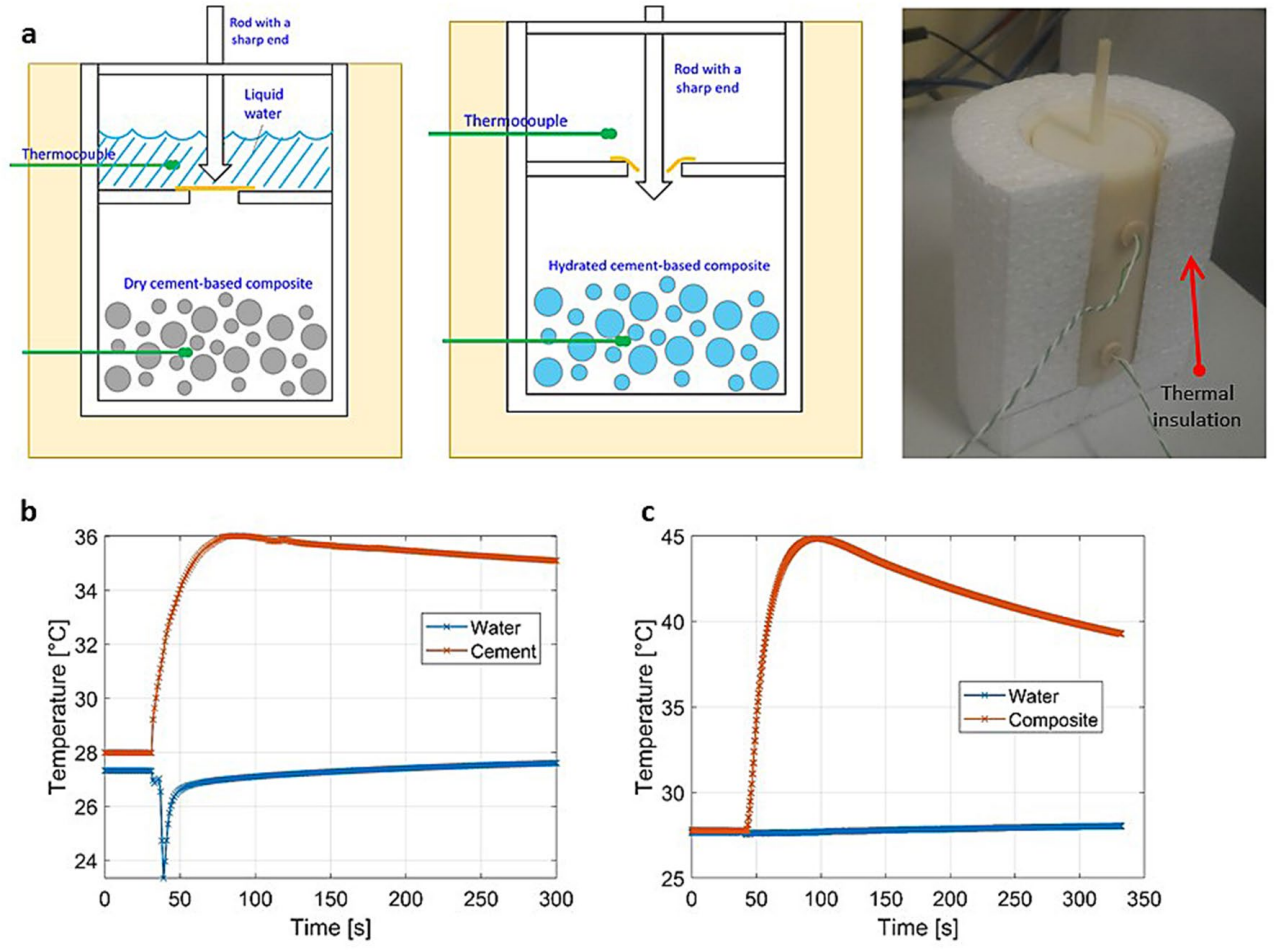
Experimental apparatus for hydration of the cement-based composite samples by liquid water. (**a**) A well insulated 3D printed vessel initially holds liquid water (top compartment) and dry sorbent samples (bottom compartment). Water and sorbent are abruptly put into contact and the temperature in the two compartment monitored. Bottom: Temperature evolution with time in the two compartment for dry cement (**b**) and cement-based composite (**c**). We notice that the temperature lift of dry cement demonstrates that the cementitious matrix itself contributes significantly to heat release. This is due to the extensive prior oven drying at 180 °C. Energy release can be estimated according to Eq. 11.

As visible in the upper part of Fig. 3, the experimental apparatus is composed of a well insulated vessel initially holding both the cement-based sorbent and liquid water into two compartments separated by a polyethylene film that can be abruptly pierced by a sharp ended rod to start sorbent hydration. Two thermocouples in contact with the sorbent and water record the temperature values in the two compartments and are used to assess when thermal equilibrium is established. At the bottom of Fig. 3, we show the typical temperature evolution of dried pure cement and cement-based composite when hydrated with liquid water. In the case of composite samples, measurements show a temperature increase of about 17 °C with an energy density $E_s=0.074$ GJ/m^3^ estimated according to Eq. 11 and assuming a sorbent mass density of 1.13 g/cm^3^ as calculated above.

It is worth stressing that the above released heat is not representative of real operating conditions, since thermochemical energy storage materials are generally operated with water vapour. Nevertheless, consistency of the aforementioned calorimetric results were also cross-checked with the values derived from water physisorption analysis reported below. A discussion of the methods used to perform such a comparison is provided in the section below.

### Gas-volumetric water vapour physisorption analysis

The gas volumetric water vapour physisorption analysis has been performed on selected specimens, and equilibrium sorption isobars at $p=12.3$ mbar (corresponding to 10 °C of evaporation temperature) were measured. All details on the utilised experimental apparatus have been provided in the methodological section below. Furthermore, the experimental water adsorption results were theoretically extrapolated in a wider range of vapour pressure values (i.e. from nearly $p=8$ mbar up to $p=200$ mbar) by assuming validity of the Dubinin–Astakhov (DA) model^23^ expressed in terms of the following Polanyi-Dubinin potential^24,25^:

6 $$\begin{aligned} A_{PD}=R T \ln {p_s(T)/p} \end{aligned}$$

with *R* and $p_s(T)$ being the universal gas constant and the vapour pressure at temperature *T*, respectively.

**Figure 4:**
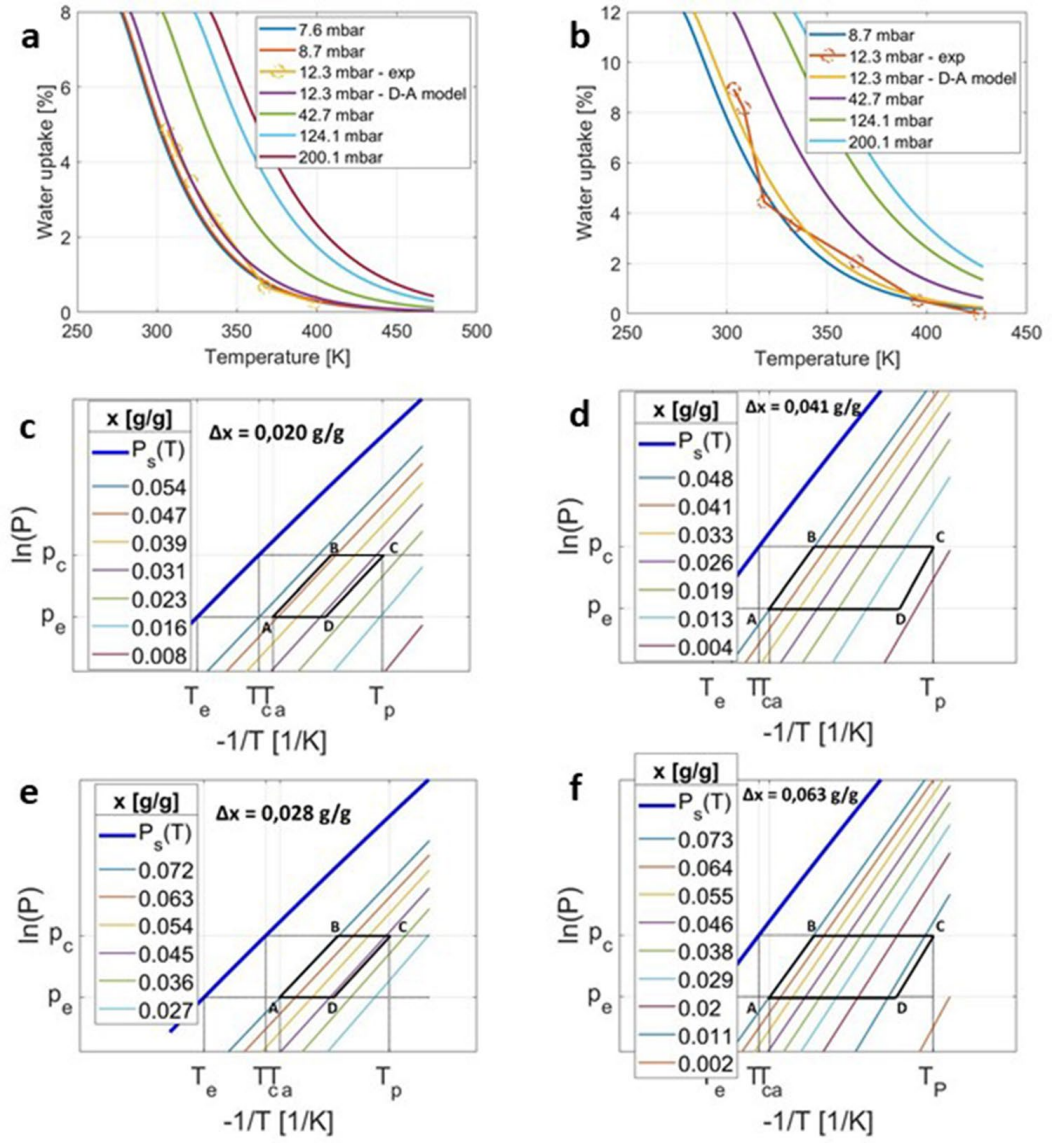
Top: (**a**, **b**) The equilibrium adsorption isobars are first experimentally assessed for two different samples with different adsorption capacity at 12.3*mbar* (symbols) and then theoretically extrapolated by using a Dubinin–Astakhov model (lines). Middle: Ideal adsorption cycles are reported for hypothetical seasonal thermal energy storage applications with maximum charging temperature of (**c**) $T_p=80 \, ^{\circ }\hbox {C}$ and (**d**) $T_p=140 \, ^{\circ }\hbox {C}$, respectively. Computations are here referred to the sample with the lowest adsorption capacity. Bottom: Ideal adsorption cycles are reported for hypothetical seasonal thermal energy storage applications with maximum charging temperature of (**e**) $T_p=80 \, ^{\circ }\hbox {C}$ and (**f**) $T_p=140 \, ^{\circ }\hbox {C}$ respectively. Computations are here referred to the sample with the largest adsorption capacity.

In the upper part of Fig. 4, we report two experimental adsorption isobars (circles) as obtained by two different specimens along with the corresponding Dubinin–Astakhov model predictions (continuous line at $p=12.3$ mbar).

7 $$\begin{aligned} \frac{x}{x_0} = \exp { \left[ - \left( \frac{A_{PD}}{E}\right) ^{n} \right] } \end{aligned}$$

where the best-fit DA parameters values were found to be $x_0=8$, $E=7000$ J/mol and $n=1.2$ for the specimen with the lowest water sorption capacity (upper-left sub-panel in Fig. 4), whereas $x_0=12$, $E=7000$ J/mol and $n=1.2$ for the specimen with the highest water sorption capacity (upper-right sub-panel in Fig. 4). The above disparity on water sorption capacity is not surprising as the synthesised composite sample, although globally homogeneous, presents an unavoidable intrinsic local heterogeneity. The aforementioned local heterogeneity clearly affects the water vapour sorption measurements that are carried out on much smaller specimens as compared to the initially synthesised sample visible in the upper-right part of Fig. 1.

Moreover, we notice that the other fitting DA parameters are within acceptable ranges according to other literature works^26,27^. As far as the isosteric heat $q_{is}$ is concerned, an average value of 50 kJ/mol was found in our case. The latter value can be judged in line with other popular materials for thermal energy storage applications. For instance, Jänchen et al.^28^ found an isosteric heat in the order of 45 kJ/mol for a silica gel-water pair, whereas Cortés et al.^29^ suggest a range of approximately 54–63 kJ/mol for Zeolite 13X-water pair. Comparing the water uptake of the specific composite considered in this study with other MgSO_4_ composite materials for thermal energy storage, their values are not considerably different. In fact, while the maximum water vapour uptake for the composite synthesised in this work was of $0.12 \, g_{H_2O}/g_{comp}$, values in the order of 0.15–0.17 $g_{H_2O}/g_{comp}$^30,31^ have been reported for composites based on Zeolite 13X and MgSO_4_.

It is now possible to perform a consistency check between the isosteric heat values and the results obtained in the above paragraph by the preliminary calorimetric analysis. During the preliminary thermal analysis, liquid water was used to hydrate the sorbent material with part of the developed heat needed to evaporate the adsorbed water molecules: As such, only the excess heat (with respect to the enthalpy of evaporation) was observed through the temperature increase.

In particular, the energy $E_s$ which causes an increase in the composite temperature upon hydration is the difference between the isosteric heat of adsorption $q_{is}$ and the enthalpy of vaporisation of water $\Delta H_{vap}$ (40.8 kJ/mol^32^), namely ca. 9.2 kJ/mol. Upon full water hydration of the sorbent (i.e. $\Delta x_{max} = 0.12 \, g_{H_2O}/g_{comp}$), the developed heat can be estimated as:

8 $$\begin{aligned} E_s=\frac{( q_{is}-\Delta H_{vap} ) \Delta x_{max}}{PM_{H_2O} } \rho _{comp} \end{aligned}$$

with $PM_{H_2O}$ being the molecular weight of water. The above computation leads to an estimate of the energy density of ca. $E_s=0.07$ GJ/m^3^, which is in line with the value obtained by the above preliminary thermal analysis (i.e. $E_s=0.074$ GJ/m^3^).

### Theoretical thermal energy storage cycle and stability analysis

Under fixed operating conditions, it is possible to theoretically estimate one of the most important figures of merit for closed adsorption plants, namely (material based) energy density. We will refer here to a possible use of cement-based composite—water pair in a seasonal thermal energy storage system for space-heating, where the discharged heat is requested at a minimum temperature $T_a$ of 35 °C. Winter temperature $T_e=10\,^{\circ }\hbox {C}$ and summer temperature $T_c=30\,^{\circ }\hbox {C}$. Before proceeding further, it is worth highlighting that the operating conditions are selected for illustration purposes only. Although the above values are reasonably chosen based on average temperatures in Mediterranean area during January and July for underfloor heating applications, we are aware of the wide parameter variability in real case heat storage and space heating systems. Therefore, the analysis reported below only serves to provide a reasonable estimate of the expected performance of the considered plants with the suggested new composite material under ideal conditions. Specifically, two different scenarios are taken into account for the highest temperature $T_p$ in the ideal thermodynamic cycle:

- $T_p = 80\,^{\circ }\hbox {C}$ will be considered representative of a cycle charged by standard flat-plate solar collector;
- $T_p = 140\,^{\circ }\hbox {C}$ will be considered representative of concentrated solar collector (e.g. Fresnel, Parabolic trough) or alternatively of a medium temperature waste heat (e.g. from industrial origin).

In the mid and bottom sub-panels of Fig. 4, we report the ideal thermodynamic cycles in the standard Clapeyron chart corresponding to the two scenarios presented above (i.e. charging by standard flat-plate collectors on the left-hand side and evacuated-tube collectors on the right-hand side). In each cycle, the water load variation $\Delta x \, [g_{H_2O}/g_{comp}]$ is evaluated, and as expected, an increase in $T_p$ implies an increase in $\Delta x$. On the basis of the $\Delta x \, g_{H_2O}/g_{comp}$ and the estimated isosteric heat $q_{is}$, the cycled thermal energy released during water adsorption follows according to Eq. 13.

In the best case scenario (i.e. sample with maximum 12 wt% water uptake) the theoretical energy density $E_v$ (based on sorbent material only) amounts to 0.088 GJ/m^3^ and 0.20 GJ/m^3^ for $T_p=80\,^{\circ }\hbox {C}$ and $T_p=140\,^{\circ }\hbox {C}$, corresponding to 0.078 GJ/ton and 0.18 GJ/ton in Table 1, respectively. On the contrary, in the worst case (i.e. sample with 8 wt% maximum water uptake), $E_v$ equals to 0.063 GJ/m^3^ and 0.13 GJ/m^3^ for $T_p=80\,^{\circ } \hbox {C}$ and $T_p=140\,^{\circ }\hbox {C}$, corresponding to 0.056 GJ/ton and 0.12 GJ/ton in Table 1, respectively. We note that those estimates of the energy density values are all based on the in situ composite sample density of 1.13 g/cm^3^ calculated above.

**Figure 5:**
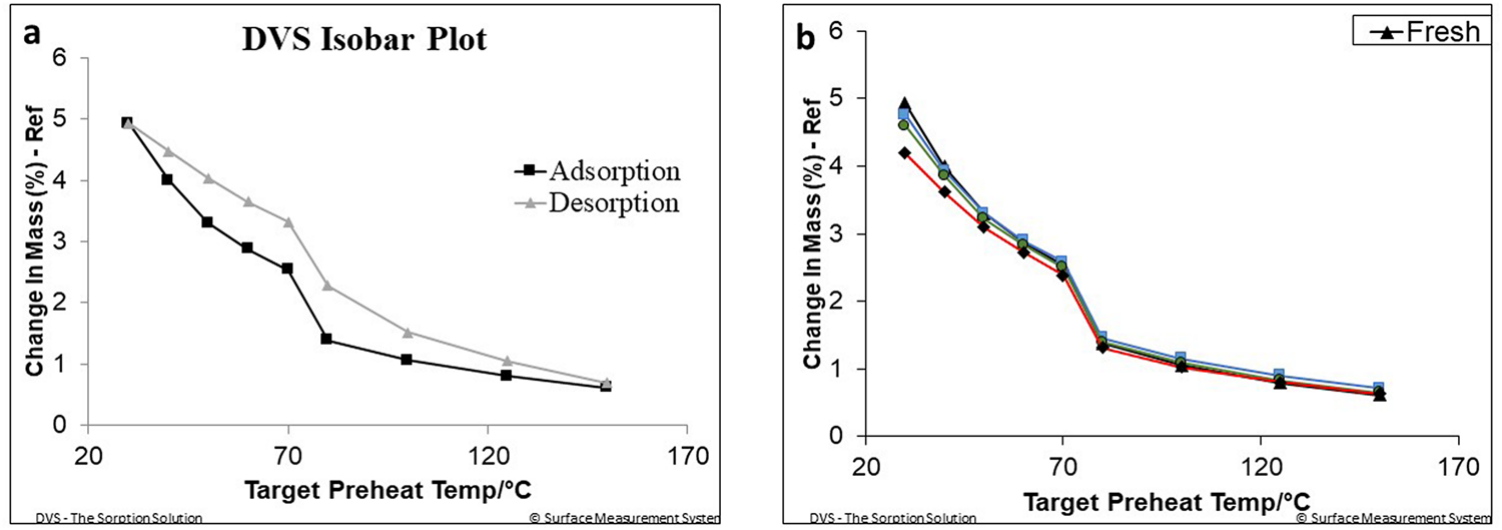
(**a**) Adsorption and desorption isobars thermogravimetrically measured at 12.3 mbar on the fresh sample; (**b**) Comparison among adsorption isobars, measured again at 12.3 mbar, at four different degrees of ageing, namely, fresh sample, 50 cycles, 110 cycles and 300 cycles.

Finally, for experimentally assessing stability in adsorption capacity upon cyclability, some of the samples have undergone several adsorption/desorption cycles with the equilibrium adsorption isobars recorded after 50, 110 and 300 cycles. While on the left-hand side of Fig. 5, we report the adsorption and desorption branches of the 8 wt% water uptake specimen, on the right-hand side of Fig. 5 a comparison of adsorption isobars (at $p=12.3$ mbar) of aged specimens is visible. We found that the hydro-thermal aging does not significantly affect the adsorption capacity of the sample. In particular, the sample aged for 300 cycles shows only a slight reduction of the adsorption capacity (within 15% as compared to the fresh sample). The latter result is encouraging, especially considering the fact that at least in seasonal TES systems, the number of ageing cycles is expected to be less than 30 for the whole lifetime of the storage (corresponding to 30 years lifetime).

### Cost analysis

An important Key Performance Indicator (KPI) useful to compare sorbent materials for TES applications is the price per stored kWh [€/kWh]. In order to calculate it, an estimate of the cost for the synthesis of the proposed composite material is needed. For pure cement powder, a maximum price of 155 €/ton is assumed, that is the highest European average factory gate price (UK, France), according to a EU report on the competitiveness of the European cement sector. The minimum price is assumed as 80 €/ton, that is the average value for other countries (Italy, Poland)^33^.

Concerning MgSO_4_-based composites, the cost is calculated as follows:

9 $$\begin{aligned} C_{comp} = \frac{c_{salt} M_{salt} + c_{cem} M_{cem} + c_{w} M_{w*}}{M_{comp}} \end{aligned}$$

where $c_{salt}$, $c_{cem}$ and $c_{w}$ are MgSO_4_ 7H_2_O, cement powder and water prices in €/ton respectively, with $c_{salt} = 77$ €/ton^3^; the price of water has very little influence and it was neglected in the calculations. $M_{salt}$, $M_{cem}$ and $M_{w*}$ are salt, cement ad water masses used for the composites synthesis, respectively. The $M_{w*}$ quantity is calculated from cement using the w*/c ratio of 0.23, the stoichiometric ratio, i.e. the water that remain in the composite, inside hydrated cement, after drying at high temperature. $M_{comp}$ is the dried composite mass, i.e. the sum of $M_{salt}$, $M_{cem}$ and $M_{w*}$. *C* is expressed in €/ton. Finally, the composite cost resulted to be $C^{max}_{comp} = 132.6$ €/ton for the highest cement price (155 €/ton) and $C^{min}_{comp} = 84.3$ €/ton for the lowest cement price (80 €/ton).

The price per stored kWh [€/kWh] can be calculated as:

10 $$\begin{aligned} KPI = \frac{C}{Q_u} \end{aligned}$$

with $Q_u$ being expressed in [kWh/ton].

For the sake of comparison, Table 1 reports the outcomes of the previously mentioned calculations for the materials analyzed in this work, along with literature data of other currently-studied materials for thermal energy storage. The salt percentage (if any) is also reported, together with the desorption temperature ($T_p$) adopted. All the literature materials listed were studied for space heating, as well. The cement-based composite samples, at maximum 12 wt% water uptake, corresponding to $T_p = 140\,^{\circ }\hbox {C}$, presents a specific density which is half the one of Zeolite 13X, and roughly one third the energy density of Zeolite 13X/MgSO_4_ composites. On the other hand, the cost of a cement-based composites is significantly lower than zeolite 13X - based materials (ca. 132 €/ton versus over 2000 €/ton). As compared to vermiculite/CaCl_2_ composites, cement-based composites present a lower energy density value (even considering $T_p = 140\,^{\circ }\hbox {C}$) and a higher specific cost. Therefore, upon optimization of the proposed cementitious composite KPIs, vermiculite based composites have been identified as the reference materials to compare with in the near future.

In this respect, also taking inspiration from other vermiculite-salt composites^34^ one possible research effort in the near future shall focus on the increase of salt content in the proposed cementitious composite.

In conclusion, although the current sub-optimal cement-based composite at $T_p = 140\,^{\circ }\hbox {C}$ presents a higher specific cost compared to vermiculite/CaCl_2_ composites, we already found a significantly lower KPI than pure zeolite 13X and zeolite 13X/MgSO_4_ composites, which are still considered among the most promising materials for thermochemical energy storage^3^. More details about estimated KPIs compared to relevant data from the literature are reported in the following Table 1.

**Table 1:** Comparison of the cement-based sorbent material presented in this study with other sorbents from the literature in terms of energy density and specific cost of the stored energy. See the text for more details.

Materials	Salt wt. (%)	$T_p \, (^{\circ }\hbox {C})$	$E_v$ (GJ/ton)	*C* (€/ton)	KPI (€/kWh)	References
C1.0-SSS (best)	21	80	0.078	132.6	3.9–6.1	
		140	0.18		1.7–2.7	
C1.0-SSS (worst)	21	80	0.056	84.3	5.5–8.6	
		140	0.12		2.7–4.2	
Zeolite 13X		180	0.54	2000	13.42	^35^
		160	0.39		18.52	
Vermiculite/CaCl_2_	57.3	85	1,00	329	1.18	^36^
Vermiculite/LiCl	59	85	2.60	4761	6.59	^37^
Zeolite 13X/MgSO_4_	10–25	150	0.65	1805	10.03	^35^
SAPO-34		95	0.73	200000	985.22	^35^
AIPO-18		95	0.87	200000	823.05	^35^
Silica gel/CaCl_2_	33.7	90	0.48	3483.50	26.39	^35^

## Discussion

In this work, we have synthesized and characterized a new composite material for thermal energy storage (or, more in general, adsorptive heat transformations). The main idea behind this study is the adoption of a widely used, easily accessible and low-cost material, such as cement paste, as a possible matrix host for accommodating hygroscopic salt-hydrates. The new in situ synthesis proposed in this study aims at a direct production of sorbents by properly mixing the cement paste with a salt-containing aqueous solution. Interestingly, relying upon the several possible salt-hydrates for thermal storage applications as well as on the large number of available agents and additives for cement preparation, the proposed approach may lay the foundations for an entire new class of composite sorbents. Clearly, it is not conceivable to explore in a single work such a huge number of possible options. Hence, without a lack of generality, here we have specifically focused on magnesium sulfate within a class G cement. A first interesting aspect of this new composite that has been analysed is the possibility of tailoring the material porosity to a good extent by controlling the water-to-cement ratio (*w*/*c* ratio). By means of a calorimentric and equilibrium water vapour physisorption analysis we have estimated an isosteric heat value in the order of 50 kJ/mol with maximum adsorption water load being in the range of 0.08–0.12 $\hbox {g}_{H_2O}/\hbox {g}_{comp}$. Above results lead to a (material based) energy density in the range of 0.088–0.20 GJ/m^3^ (for an ideal closed thermal energy storage cycle and considering the best tested sample). The estimated energy density is significantly lower than the one reported in the literature for other composites. Nevertheless, the comparison with more standard materials becomes significantly more favorable when considering the specific Key Performance Indicator—KPI—of interest in this work, namely the specific cost of the stored energy. At the same time, we stress that the presented results refer to samples that have not been optimized whatsoever. Indeed, as visible in Table 1, vermiculite-based composites still show better performance with respect to all figures of merit. Hence, we are aware that an extensive optimization is needed in the near future in order to significantly increase the salt content into the cementitious matrix. This will possibly lead to materials with much higher water load adsorptions (i.e. $> 0.20 \, g_{H_2O}/g_{comp}$). Nonetheless, an encouraging aspects that emerges from our study is the negligible variation in water sorption capability of the tested material samples after one hundred water soprtion-desorption cycles. Moreover, the ageing test under sorption-adsortion cycles is a first important indication, however more studies are needed to assess the material behaviour after months or years (i.e. under time ageing). Finally, all the reported results are valid under equilibrium conditions and future studies have to include also kinetics and analyze the material performance during dynamic operation as well.

## Methods

### Preparation of cement composites containing salt

For the synthesis of highly-porous pure cement samples, class G cement (Lafarge North America) was used. Table 2 shows the typical composition and properties of Class G cement.

**Table 2:** Typical composition and properties of Class G cements.

Oxide	[wt.%]	Phase	[wt.%]
${\hbox {SiO}}_2$	21.7	C3S	66
CaO	62.9	C2S	18
${\hbox {Al}}_2 {\hbox {O}}_3$	3.2	C3A	7
${\hbox {Fe}}_2 {\hbox {O}}_3$	3.7	C4AF	9
MgO	4.3		
${\hbox {SO}}_3$	2.2		
${\hbox {Na}}_2 \hbox {O}$	0.54		
Loss on ignition	0.74		
Insoluble residue	0.14		

The samples of hydrated cement without salt were prepared at different water-to-cement (*w/c*) ratios, in order to estimate the relationship between *w/c* and the porosity of the hydrated cement. Table 3 shows the quantity in grams to obtain the different w/c ratio samples. In the case of the in situ composite synthesis, instead, the chosen *w/c* ratio was 1. The hydrated cement preparation consists in weighing the correct amount of water and cement, and in mixing both with a rotational mixer for 60 s in order to obtain a homogeneous paste. Then the cement paste is poured slowly into molds, that are placed inside an oven at 85 °C and 100% relative humidity for 24 h, in order to complete the hydration process.

**Table 3:** Mix design for the preparation of highly porous cement.

w/c	Water (g)	Cement (g)
0.5	20.7	41.4
1	41.4	41.4
1.5	62.1	41.4
2	82.8	41.4

In the case of the in situ process, the procedure is very similar, but instead of using distilled water, a solution obtained dissolving 13.4 g of MgSO_4_ salt in 41.4 g of distilled water was used for the preparation of the sample. The salt and water are weighed and mixed up to complete dissolution of the salt, then cement powder is mixed and the process continues as in the case of pure cement. Given the correlation between high energy density and relatively low cost, MgSO_4_ 7H_2_O (Merck) was chosen as the salt investigated in this study. To avoid any error in the *w/c* ratio, since magnesium sulfate is typically supplied as MgSO_4_ 7H_2_O, in the calculation of salt content only the anhydrous salt weight was considered, while the hydration water was calculated as part of the total water used in the preparation.

### Characterization of the material

The density of samples was measured by measuring weight and dimension of cement parallelepipedal blocks, with polished surfaces in order to reduce the error due to surface roughness. The weight was measured with a Orma analytical balance (Model BC, accuracy 0.1 mg) and the dimensions of the block by Mitutoyo digital caliper. The morphological characterization was performed by means of an optical microscope (Leica CTR 4000 inverted microscope). Observation were performed both on the external surface and on the cross-section of specimens. The mechanical characterization was performed with a Zwick Z050 testing machine, equipped with a 50 kN load cell. The volumetric nitrogen adsorption/desorption isotherms were carried out using a Micromeritics Tristar II instrument. Pure cement as well as the in situ composite were analyzed. Before performing the measurements, the samples were dried at 100 °C, for ca. 3 h, and outgassed until reaching the desired level of vacuum prior to analysis. The pore volume and size distribution were evaluated by applying the Barrett–Joyner–Halenda model (BJH)^38^, a commonly used method to determine the pore structure in mesoporous materials.

### Preliminary calorimetric analysis

A preliminary calorimetric analysis is performed by the experimental apparatus shown in Fig. 3. In a well insulated vessel, liquid water is poured onto the cement-based composite sorbent (obtained upon oven drying at 180 °C for 5 h) and the temperature increase recorded during the hydration process. Firstly, the dried sorbent sample and liquid water are held into separated (by a polyethylene film) compartments. The test starts after a sufficient time period has elapsed so that thermal equilibrium is established between liquid water and sorbent material. Hence, the polyethylene film is abruptly pierced by a sharp ended rod and water poured instantaneously on the underneath composite sample. Water-composite system temperature is recorded continuously over time by a thermocouple, to acquire both the potential $\Delta T$ produced and the time interval needed to reach the maximum temperature.

The vessel compartments were designed to accommodate a maximum of ca. 35 g of cement-based composite and the corresponding hydration water with an overabundant ratio of 0.7 (i.e. ca. 24 g). The vessel has been 3D printed in ABS (acrylonitrile butadiene styrene) and made waterproof by a colloidal agent spread over the vessel external surface.

Two K-type thermocouples (Chromel (Ni-Cr) (+) /Alumel (Ni-Al) (-)) were adopted to monitor the temperature evolution into the two vessel compartment. Data acquisition was performed using the National Instruments acquisition system and the LabVIEW 2017 software.

The temperature difference $\Delta T$ between the initial and the maximum recorded temperature was used to estimate the composite energy density, by means of the following equation:

11 $$\begin{aligned} \Delta T \sum _{i} M_i c_{p,i} = E \end{aligned}$$

where

- $M_i$ is the mass of each component in the system (i.e. pure cement matrix, water and salt hydrate).
- $c_{p,i}$ is the *i*-th specific heat capacity, with the used values being: 4.816 kJ/kg/K for water, 1.44 kJ/kg/K for pure cement, and 1.58 kJ/kg/K for MgSO_4_ 7H_2_O^39^.

It is worth stressing that Eq. 11 is only valid under adiabatic conditions of the vessel. In fact, the right-hand side of Eq. 11 should more rigorously include an additional term accounting for the thermal losses towards the environment. The latter term depends on the overall thermal transmittance of the vessel and its computations should be conducted under transient conditions. However, we have estimated that the amount of energy lost towards the environment during the test time period is a small fraction of the adsorption energy and it can be safely neglected.

### Thermo-gravimetric water vapour sorption analysis

Thermo-gravimetric dynamic water vapour sorption analysis was performed to assess equilibrium sorption isobars. The experimental setup is based on a DVS-Vacuum analyser, able to measure sorption equilibrium with an accuracy of $0.1 \, \mu \hbox {g}$, either under isobaric or isothermal conditions, in a wide range of temperatures (from room temperature up to 400 °C) and relative pressures (from 0 up to 0.95).

Once the sample is put inside of the testing chamber, the specimen undergoes a preliminary desorption process (at evacuated conditions and 150 °C for 8 h), to assure its complete dehydration. Afterwards, the connection with evaporator opened. The target water vapour pressure is imposed dynamically, thanks to a slow water vapour flow controlled by a butterfly valve, modulating the suction effect of the vacuum pump. In this way, the equilibrium in each step is achieved more rapidly comparing to typical sorption under static water vapour conditions. Hence, water uptake ($g_{H2O}/g_{comp}$) at different equilibrium points is acquired, by means of sample mass difference with respect to the initial condition set at 150 °C and under continuous evacuation. Equilibrium is reached when the sample weight does not change for 1 h. During the measurements, water vapour pressure is kept constant. Once the measure is complete, a water uptake versus temperature curve is obtained, at a given water vapour pressure. By repeating the measures at different water vapour pressures, it is possible to obtain different sorption isobars in the water uptake—temperature chart, as shown in Fig. 4. A key quantity obtainable from the Clausius-Clapeyron relation is the isosteric heat of sorption $q_{is}$. It depends on the sorbent-adsorbate system and is expressed as follows at a fixed load:

12 $$\begin{aligned} q_{is} = R \frac{\partial {\left( \ln {p} \right) }}{\partial {\left( -1/T \right) }} \end{aligned}$$

where *R* is the gas constant (8.314 J/mol/K) and the derivative represents the isosteres slope in the Clapeyron chart.

In a sorption cycle, the heat released during the discharge phase (referred to as cycled heat $Q_u \, [kJ/kg_{comp}]$) is related to $q_{is}$ as follows:

13 $$\begin{aligned} Q_{u} = \frac{q_{is} \Delta x }{ PM_{H_2O}} \end{aligned}$$

where $\Delta x$ is the amount of water vapour adsorbed ($[g_{H_2O}/g_{comp}]$) and $PM_{H_2O}$ is water molar mass.

### A protocol for characterizing hydrothermal stability

The experimental ageing setup used in this work has been described elsewhere^40^. It consists in a vacuum chamber and in a glass evaporator/condenser; inside the vacuum chamber, three plate heat exchangers (HEX) are located to define the ageing conditions of the samples. In particular, the samples can be aged either under cycling conditions (i.e. varying the temperature) or under shelf conditions (i.e. keeping the temperature constant); the evaporator/condenser is able to maintain a constant water vapor pressure inside the system. The experimental plant is equipped by three thermo-cryostats in order to: thermostat at a constant temperature the refrigerant inside the evaporator/condenser, provide high temperature for desorption phase and low temperature for adsorption step. A completely automatic hydraulic system permits to mimic the cycle boundary conditions. The system was equipped by temperature and pressure sensors in order to control and record the main thermo-physical parameters that affect the ageing tests. The management and acquisition system is based on National Instrument Compact Field Point hardware and a LabView control panel, which permits both automatic and manual management of the testing rig as well as the acquisition of all the relevant parameters (i.e. temperature and pressure). Lastly, the testing rig is located inside the thermostatic box in order to avoid the condensation of water vapor on the internal surface of the vacuum chamber when the ambient temperature is lower than the temperature set in the evaporator/condenser. In the present work, only the cycling ageing conditions were applied to the samples. The protocol defined to verify the hydrothermal stability of the sample consists in the following cycling conditions: $T_{ads} = 35\,^{\circ }\hbox {C}$; $T_{des} = 90\,^{\circ }\hbox {C}$; $T_{ev} = 7\,^{\circ }\hbox {C}$ (1.05 kPa); $t_{ads} = t_{des} = 10\,\hbox{min}$.

The parameters of $T_{ads}$, $T_{des}$ and $T_{ev}$ were chosen in order to simulate realistic conditions under which the storage operates, while, the choice of the duration of the adsorption and desorption phases ($t_{ads} = t_{des}$) was selected to guarantee sufficient time for the adsorption/desorption phases while accelerating the aging of the sample. In this way, about 50 cycles per day were performed.

Different aluminum sample pans, containing the cement composite, were inserted in the aging testing rig. To insure a good heat transfer between the plate HEX and sample pans, thermal conductive paste was placed between the sample pans and the HEX. The choice to use different sample holders resulted from the need to ensure an intimate contact between the material and the heat exchanger and a homogeneous distribution of the water vapor inside the sample. Therefore, by distributing a thin layer of sample in different pans, it was possible to ensure both the homogeneous distribution of the water vapor and the correct heat transfer towards the material.

The cement composite was aged for 300 cycles. Three samplings were done, precisely at 50, 110 and 300 cycles.

In order to verify the stability of the aged cement composite sample, the measurement of equilibrium curve in isobaric condition was carried out by means of DVS Vacuum analyzer, provided by Surface Measurement Systems. To evaluate the equilibrium properties the following testing procedure was established: around 10–15 mg of cement composite was put on the high-resolution microbalance, the material was degassed under continuous evacuation at 150 °C for 360 minutes at absolute pressure 1 10^−7^ Pa. Then, the equilibrium was calculated at 30, 40, 50, 60, 70, 80, 100, 125 and 150 °C under isobaric condition at pressure of 1.05 kPa (i.e. 7 °C in the evaporator).
